# A Reactivity-Based ^18^F-Labeled Probe for PET Imaging of Oxidative Stress in Chemotherapy-Induced Cardiotoxicity

**DOI:** 10.1021/acs.molpharmaceut.1c00496

**Published:** 2021-11-30

**Authors:** Filipa Mota, Victoria R. Pell, Nisha Singh, Friedrich Baark, Edward Waters, Pragalath Sadasivam, Richard Southworth, Ran Yan

**Affiliations:** § School of Biomedical Engineering & Imaging Sciences, King’s College London, King’s Health Partners, St Thomas’ Hospital, London SE1 7EH, United Kingdom; † Department of Neuroimaging, Institute of Psychiatry, Psychology, and Neuroscience, King’s College London, London SE5 8AF, United Kingdom

**Keywords:** reactive oxygen species, oxidative stress, cardiotoxicity, PET imaging, fluorine-18

## Abstract

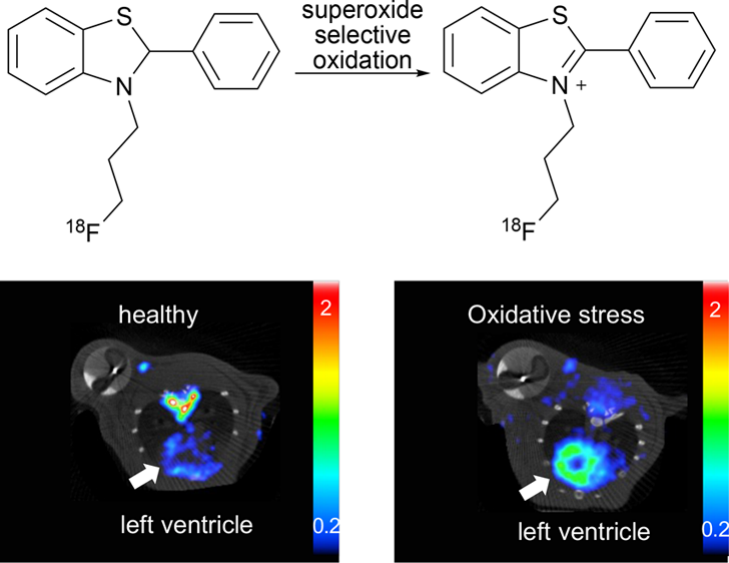

Oxidative stress underlies the pathology of many human diseases, including the doxorubicin-induced off-target cardiotoxicity in cancer chemotherapies. Since current diagnostic procedures are only capable of monitoring cardiac function, a noninvasive means of detecting biochemical changes in redox status prior to irreversible functional changes is highly desirable for both early diagnosis and prognosis. We designed a novel ^18^F-labeled molecular probe, ^18^F-FPBT, for the direct detection of superoxide *in vivo* using positron emission tomography (PET). ^18^F-FPBT was radiosynthesized in one step by nucleophilic radiofluorination. *In vitro*, ^18^F-FPBT showed rapid and selective oxidation by superoxide (around 60% in 5 min) compared to other physiological ROS. In healthy mice and rats, ^18^F-FBPT is distributed to all major organs in the first few minutes post injection and is rapidly cleared via both renal and hepatobiliary routes with minimal background retention in the heart. In a rat model of doxorubicin-induced cardiotoxicity, ^18^F-FBPT showed significantly higher (*P* < 0.05) uptake in the hearts of treated animals compared to healthy controls. These results warrant further optimization of ^18^F-FBPT for clinical translation.

## Introduction

Reactive oxygen species (ROS) are generated as normal by-products of metabolism in the electron transport chain and play an integral role in the regulation of cell growth, neurotransmission, and the immune response.^1^ However, uncontrolled ROS production leads to the oxidation of DNA, proteins, and lipids, underlying the pathogenesis of many cardiovascular and neurodegenerative diseases, as well as cancers and inflammatory conditions.^2−7^ In the cardiovascular system, elevated ROS are responsible for tissue injury during ischemia/reperfusion and have been linked to the progression from cardiac hypertrophy to heart failure, the evolution of atherosclerotic plaques, and the cardiac and microvascular dysfunction associated with diabetes. ROS production has also been linked to the cardiotoxicity of cancer chemotherapeutic agents, which severely limits their dosimetry and effectiveness.^8−11^ Most notably, the cardiotoxicity induced by doxorubicin, a widely used cancer chemotherapy agent, has been linked to ROS generation.^12,13^

A clinically translatable means of noninvasively identifying and quantifying elevated ROS production *in vivo* would be highly desirable for both diagnostic and prognostic purposes and the development and evaluation of emerging cardioprotective approaches.^14^

Several radiolabeled small molecules have been reported to indirectly detect oxidative stress by positron emission tomography.^15,16^ Radiotracers based on the fluorescent probe dihydroethidium have been tested in rodent models of superoxide-associated cardiotoxicity and neuroinflammation.^17,18^ While some of these designs are promising, none have yet progressed to clinical evaluation, and the search for optimal ROS-sensing PET radiotracers continues.

**Figure 1:**
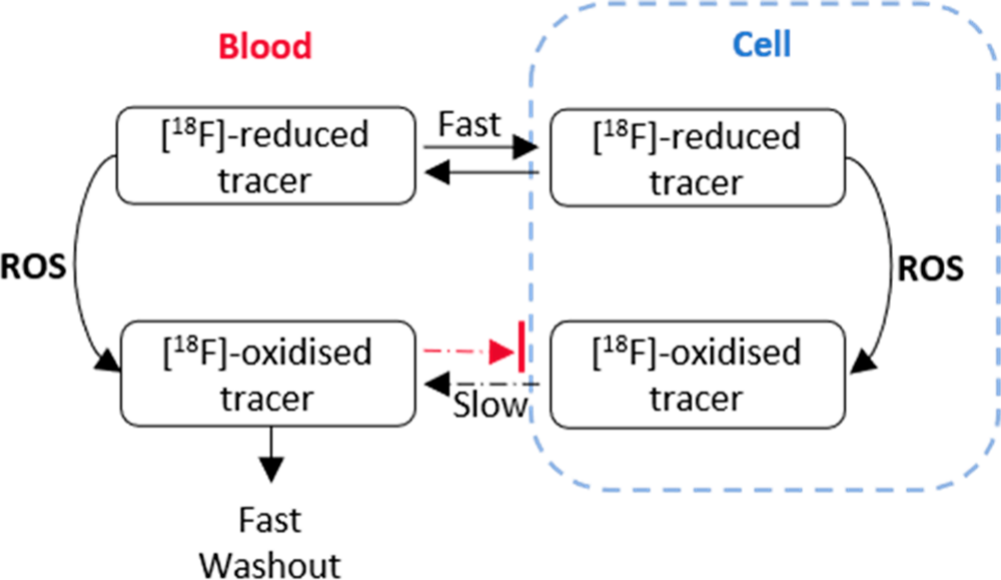
Proposed mechanism of PET tracer, ^18^F-FPBT, for the direct detection of intracellular reactive oxygen species.

Here, we describe a novel chemical scaffold with potential utility as ^18^F-labeled PET tracers for the direct detection of ROS *in vivo*. Phenyl benzothiazoles were identified as promising candidates due to their low molecular weight, well-established chemistry, susceptibility to oxidation by superoxide, and physicochemical properties that are favorable to the cell membrane and blood–brain barrier permeability.^19,20^ Our tracer, [^18^F]3-(3-fluoropropyl)-2-phenyl-2,3-dihydrobenzo[*d*]thiazole, ^18^F-FPBT was designed to be sufficiently lipophilic in its reduced form to facilitate cell membrane and blood–brain barrier penetration, while sufficiently hydrophilic when oxidized to become transiently retained in cells under oxidative stress to provide ROS-sensitive PET contrast (Figure 1). Herein, we report the synthesis, ^18^F-labeling, *in vitro*, and preclinical *in vivo* evaluation of ^18^F-FPBT in healthy mice and rats, as well as in a rat model of doxorubicin-induced cardiotoxicity.

## Materials and Methods

### General Information

HPLC analysis was performed with an Agilent 1200 HPLC system equipped with a 1200 series diode array detector. Radio-HPLC analysis was performed with an Agilent 1200 HPLC system equipped with a series diode array detector and a Raytest GABI Star radioactivity detector. ^18^F-Fluoride was purchased from either the PET Center at St. Thomas’ Hospital or Alliance Medical UK. All reagents were purchased from Sigma-Aldrich and were used without further purification. The radiochemical yield was calculated as a percentage of purified tracer to starting activity. The radiochemical conversion was calculated based on the HPLC peak AUCs. Preclinical PET/CT images were acquired using a NanoScan PET/CT (Mediso, Budapest, Hungary) scanner. Data are represented as mean ± SD, unless stated otherwise. Chromatographs and graphs were plotted on GraphPad Prism 8.

### Chemical Synthesis

The synthesis and characterization of the nonradioactive reference compound **1**, intermediates, **2**, **3**, **4**, and radiolabeling precursor **5** is described in the Supporting Information.

### Radiosynthesis

#### Radiosynthesis of ^18^F-FPBT ([^18^F] **1**)

^18^F-Fluoride (∼1.2 GBq) in water was trapped in a QMA cartridge (Waters Sep-Pak light, pretreated with 10 mL of water) and released with 1.0 mL of a Kryptofix 222 and potassium carbonate mixture (30:15 mM) in acetonitrile/water (85:15) into a 5 mL Wheaton vial. After the solvents were removed by heating at 110 °C under a stream of nitrogen for 15 min, azeotropic distillation with anhydrous acetonitrile (400 μL) was repeated twice at 90 °C for another 15 min. A solution of **5** (6 mg, 16 μmol) in anhydrous acetonitrile (400 μL) was then added and heated at 80 °C for 15 min. The reaction was cooled to room temperature, quenched by the addition of water (100 μL), and purified by semipreparative HPLC. 3-(3-[^18^F]Fluoropropyl)-2-phenyl-2,3-dihydrobenzo[*d*]thiazole (^18^F-FPBT, [^18^F] **1**) was purified with a ZORBAX column (300SB-C18, semipreparative 9.4 mm × 250 mm, 5 μm) using acetonitrile and water as the mobile phase, at a flow rate of 3.0 mL/min. The following gradient was used: from 50% to 90% acetonitrile in 15 min; kept at 90% acetonitrile for 10 min; from 90 to 50% acetonitrile in 5 min. [^18^F] **1** has an HPLC retention time of 12.5 min. It was collected from the HPLC column and diluted to 10% acetonitrile in water. It was trapped onto a Sep-Pak C-18 light cartridge (preactivated with 5 mL of methanol followed by 5 mL of water). The cartridge was washed with 2 mL of water, and [^18^F] **1** was then eluted with 1 mL of ethanol to give [^18^F] **1** in 60 ± 20% radiochemical conversion, decay corrected isolated RCYs around 33 ± 8%, and >98% radiochemical purity (*n* = 10).

#### Radiosynthesis of ^18^F-FPBT-Ox ([^18^F] **3**)

[^18^F] **3** was obtained by reacting [^18^F] **1** (∼50 MBq, 1.0 mL in PBS containing 10% ethanol) with an excess of potassium superoxide (10 mg) until >90% oxidation was observed by radio-HPLC in about 15 min. [^18^F] **3** has a HPLC retention time of 4.5 min under the same HPLC setting as [^18^F] **1**. [^18^F] **3** was used in the same formulation as the reduced tracer [^18^F] **1**.

## *In Vitro* Characterization

### Lipophilicity

The lipophilicity of ^18^F-FPBT and ^18^F-FPBT-Ox was determined by a conventional partition method between 1-octanol and phosphate buffered saline (PBS), pH 7.4. 1-Octanol was presaturated with PBS before use. The radiotracer (∼2 KBq) was added to a mixture of PBS (200 μL) and 1-octanol (200 μL) in a 1.5 mL Eppendorf vial (*n* = 6). The mixture was vigorously agitated at rt for 5 min and then centrifuged at 3000 *g* for 10 min. A 100 μL aliquot from each layer was drawn for measurement in a gamma counter. The Log*D*_oct/PBS_ was calculated as follows: log [(cpm in the 1-octanol layer – cpm 1-octanol blank)/(cpm in the PBS layer – cpm PBS blank)].

### Vial Stability

^18^F-FPBT (1–4 MBq) in 100% ethanol or 5% ethanol in PBS containing sodium ascorbate (0.01 mg/mL) was kept at room temperature for 4 h and then analyzed by radio-HPLC.

### Serum Stability

^18^F-FPBT (∼3.0 MBq) in 5% ethanol in PBS (200 μL) containing sodium ascorbate (0.01 mg/mL) was incubated with rat serum (200 μL) at 37 °C for 1 min or 1 h. Subsequently, plasma proteins were precipitated from the supernatant with ice-cold acetonitrile (1.5 mL), and samples were centrifuged (3 min, 13 000 rpm). The residual activity in the pellet was <3%. The supernatant (∼2.0 MBq, 1.0 mL) was analyzed by radio-HPLC. HPLC method: ZORBAX column (300SB-C18, semipreparative 9.4 mm × 250 mm, 5 μm) using acetonitrile and water as the mobile phase, at a flow rate of 3.0 mL/min. The following gradient was used: from 50% to 90% acetonitrile in 15 min; kept at 90% acetonitrile for 10 min; from 90 to 50% acetonitrile in 5 min.

^18^F-FBPT-Ox (2–4 MBq, 50 μL in 10% EtOH/PBS) was added to serum (500 μL) and incubated at 37 °C for 5, 15, and 30 min, respectively. Plasma proteins were precipitated with a 5-sulfosalicylic acid water solution (10% w/v, 1 mL) and centrifuged (3 min, 13 000 rpm). The supernatant was removed and diluted with 500 μL of DI H_2_O and analyzed via radio-HPLC. A control experiment was performed where ^18^F-FBPT-Ox (2 MBq, 50 μL in 10% EtOH/PBS) was added to 500 μL of PBS, incubated at 37 °C for 30 min. A 5-sulfosalicylic acid water solution (10% w/v, 1 mL) was added, thoroughly mixed, then diluted with 500 μL of DI H_2_O, and analyzed via radio-HPLC. HPLC method: ZORBAX column (300SB-C18, semipreparative 9.4 mm × 250 mm, 5 μm). Solvent A: H_2_O. Solvent B: MeCN. Flow rate: 3.0 mL/min; 0–5 min, 0% of B; 5–20 min, 0–50% of B; 20–30 min, 50–0% of B.

### Reactivity of ^18^F-FPBT to Various Reactive Oxygen Species

^18^F-FPBT (0.5–2.0 MBq) was reacted with potassium superoxide (source of superoxide, 1 mg), hydrogen peroxide (100 μM), Fenton-reaction-generated hydroxyl radicals (100 μM), iron(II) sulfate heptahydrate (100 μM), *tert*-butyl hydroperoxide (100 μM), *tert*-butoxy radical (100 μM), and 3-morpholinosydnomine (source of peroxynitrite (100 μM)) for 5 min or diethylamine NONOate (source of nitric oxide, 100 μM) for 30 min at room temperature, after which it was subjected to radio-HPLC analysis and % oxidation was calculated.

### Animal Studies

All experiments were performed in accordance with the Animals (Scientific Procedures) Act 1986 under project license nos. PPL P96678ED7 and PPL 70/8482. Male Wistar rats were obtained from Envigo Ltd. and C57BL/6 mice from Charles Rivers. All animals went through a 7 day acclimatization period at the Biological Services Unit at St. Thomas’ Hospital prior to any experiments. ^18^F-FPBT was formulated in ∼5% ethanol in PBS, containing 0.01 mg/mL sodium ascorbate for injection. ^18^F-FPBT-Ox was formulated in ∼5% ethanol in PBS. All PET imaging studies were performed using a Nano-Scan PET/CT (Mediso, Budapest, Hungary) scanner. All PET/CT data were reconstructed with the Monte Carlo-based full-3D iterative algorithm Tera-Tomo (Mediso Medical Imaging Systems, Budapest, Hungary). All procedures were performed under 2% isoflurane in oxygen anesthesia.

### PET/CT Imaging of Healthy Mice

C57BL/6 mice (male, 25 ± 2 g, *n* = 3) were placed on the PET scanner bed and injected with ^18^F-FPBT (1.7 ± 0.4 MBq) through the tail vein at the start time of PET acquisition. A dynamic PET scan was performed for 60 min, followed by a CT scan and *ex vivo* biodistribution. The PET data was reconstructed into 5 frames of 60 s, 3 frames of 300 s, and 4 frames of 600 s. Regions of interest (ROIs) were drawn and quantified using VivoQuant software (v3.0, inviCRO, LLC, Boston, USA). Organ uptake was calculated as standard uptake values (SUV), and data are reported as mean ± SD.

### Post-Mortem Biodistributions in Healthy Rats

Wistar rats (male, 319 ± 20 g) were injected intravenously via the tail vein with either ^18^F-FPBT (1.0 ± 0.4 MBq) or ^18^F-FPBT-Ox (1.1 ± 0.5 MBq). The animals were sacrificed by cervical dislocation at 1, 5, or 30 min postinjection (*n* = 3/group). Organs and tissues of interest were harvested and weighed, and the radioactivity was measured in a gamma counter. Organ uptake was calculated as the percentage injected dose per gram of tissue mass (% ID/g). Data are reported as mean ± SD.

## Rat Model of Doxorubicin-Induced Cardiotoxicity

### Minipump Implantation

Male Wistar rats (280–300 g) were used in all experiments. Subcutaneous 7 day osmotic pumps (Alzet), containing either doxorubicin (Cambridge Bioscience, 30 mg/kg cumulative dose) or vehicle (sterile 0.9% NaCl), were inserted into rats under 2% isoflurane in 100% oxygen.

### Echocardiography

In order to assess cardiac function, all rats were subjected to a cardiac ultrasound (Vevo 770TM, VisualSonics) 1 day prior to and on day 6 of osmotic pump implantation. Rats were anesthetized with 2% isoflurane in 100% oxygen and maintained at 37 °C via a homeothermic platform and rectal thermometer. High-resolution parasternal left ventricle (LV) long axis M-mode and B-mode images were obtained using an RMV710B transducer. Images were analyzed offline using Vevo Software to determine LV function. Data are reported as mean ± SD. Statistical analysis was performed using one-way ANOVA with Tukey’s multiple comparisons test.

### PET/CT Imaging in a Rat Model of Doxorubicin-Induced Cardiotoxicity

Wistar rats that had received either doxorubicin treatment (male, 267 ± 19 g, *n* = 6) or saline (male, 303 ± 9 g, *n* = 4) via osmotic pumps for 7 days were injected with ^18^F-FPBT (2.6 ± 1.5 MBq) via the tail vein on the PET/CT scanner bed. The time of injection coincided with the beginning of PET acquisition in order to obtain dynamic tracer uptake information. PET scans with the chest area in the field of vision were acquired for 30 min, followed by a CT scan, after which the animals were sacrificed and organs harvested, weighed, and gamma-counted as for the biodistribution protocol. The hearts were frozen in liquid nitrogen for gamma counting and oxidative stress biomarker assays. The PET data was reconstructed into three frames: 0–3 min, 3–10 min, and 10–30 min. For the time–activity curve, the PET data was reconstructed into 1 min per frame in the first 5 min and then 5 min per frame between 5 and 30 min. Using VivoQuant software (v3.0, inviCRO, LLC, Boston, USA), regions of interest (ROIs) were drawn in the left ventricle and blood inside the myocardium. Radioactivity uptake in the heart was calculated as a standard uptake value ratio (SUVR) between left ventricle and blood. Data are reported as mean ± SD. Statistical analysis was performed using one-way ANOVA with Tukey’s multiple comparisons test.

### *Ex Vivo* Heart Biomarkers of Oxidative Stress

Total glutathione concentration was determined by an enzyme recycling method.^21^ Lipid peroxidation was evaluated using the lipid peroxidation (MDA) assay kit from Sigma-Aldrich, which measures the colorimetric product that results from the reaction of malondialdehyde (MDA) present in the tissue with thiobarbituric (TBA) acid. Iron content was measured in isolated mitochondria and cytosolic fractions using the iron colorimetric assay kit from BioVision following the manufacture’s protocol.

### *In Vivo* Blood Stability of ^18^F-FBPT-Ox

Wistar rats (male, 320 ± 20 g) were intravenously injected with ^18^F-FPBT-Ox (∼15 MBq). The animals were sacrificed by cervical dislocation at 30 min postinjection (*n* = 2). Blood samples were collected in heparin-coated tubes. After centrifugation (5 min, 13 000 rpm), the plasma (∼1 mL) was separated. Plasma proteins were subsequently precipitated with 5-sulfosalicylic acid water solution (10% w/v, 1.5 mL) and centrifuged (5 min, 13,000 rpm). The supernatant was removed and diluted with 500 μL of DI H_2_O and analyzed via radio-HPLC. HPLC method: ZORBAX column (300SB-C18, semipreparative 9.4 mm × 250 mm, 5 μm). Solvent A: H_2_O. Solvent B: MeCN. Flow rate: 3.0 mL/min; 0–5 min, 0% of B; 5–20 min, 0–50% of B; 20–30 min, 50–0% of B. HPLC eluent was collected every minute in 30 vials separately and submitted to gamma counting. The counts per minute in each vial were plotted against the corresponding time point.

### Statistical Analyses

Statistical analyses were performed using Graphpad Prism 9 software. Statistical comparisons between two groups were determined by unpaired *t* test with Welch’s correction, and comparisons between multiple groups were determined by one-way ANOVA with Tukey’s correction.

## Results

### Synthesis

**Figure 2:**
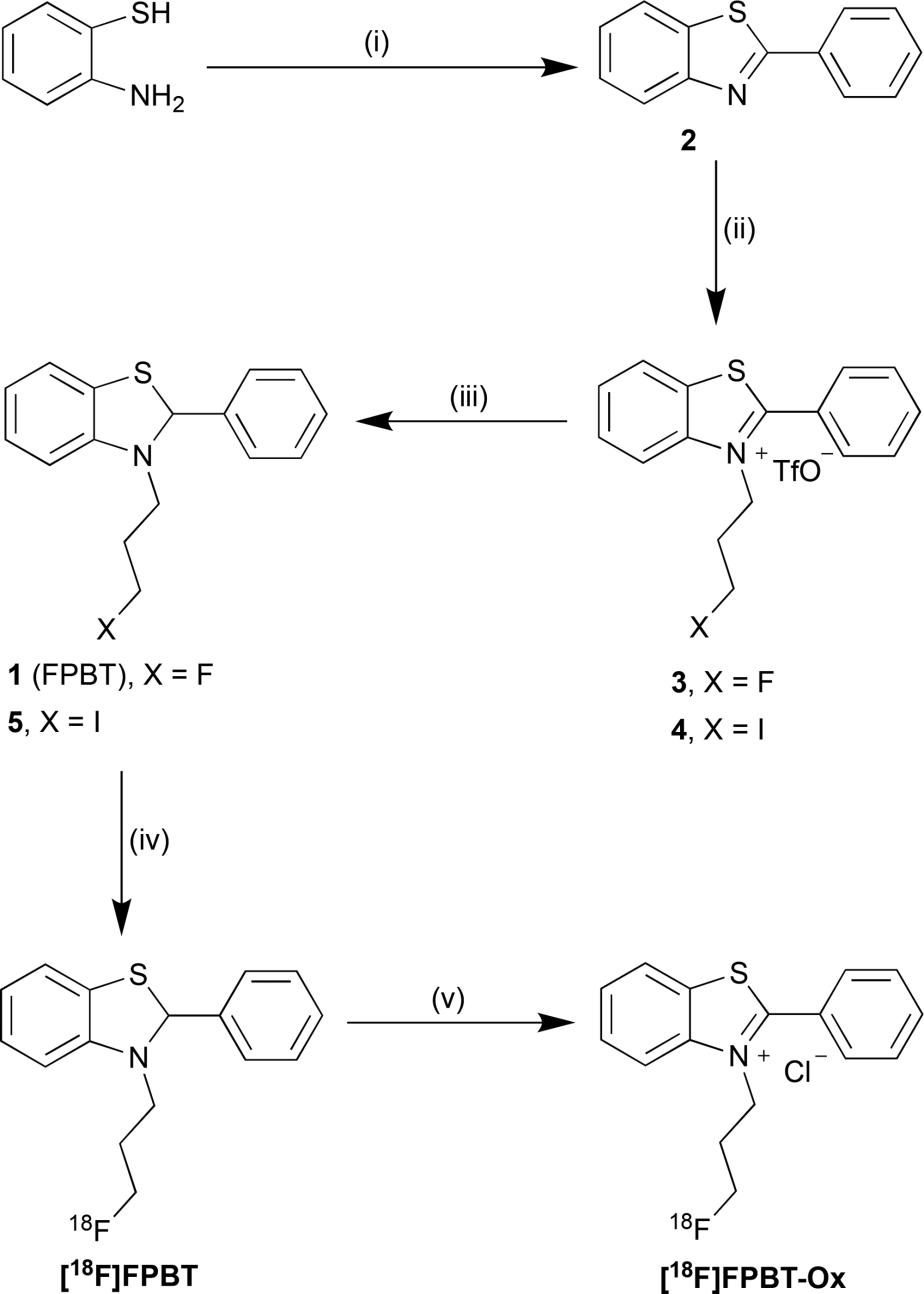
Precursor and nonradioactive reference compounds synthesis and radiosynthesis of ^18^F-FPBT and ^18^F-FPBT-Ox. Reagents and conditions: (i) benzoic acid, polyphosphoric acid, 150 °C, 24 h, 67%; (ii) 3-iodopropyl trifluoromethanesulfonate or 3-fluoropropyl trifluoromethanesulfonate, NaHCO_3_, nitrobenzene, rt, 24 h, 20% for **3** and 32% for **4**; (iii) NaBH_4_, THF, methanol, rt, 20 min, 52% for **1** and 56% for **5**; (iv) K^18^F, K_2_CO_3_, Kryptofix 2.2.2., acetonitrile, 80 °C, 15 min, RCYs 33 ± 8% (*n* = 10); (v) KO_2_, PBS, rt, 15 min, RCYs 93 ± 2% (*n* = 5).

The nonradioactive reference compound of ^18^F-FPBT and its iodinated precursor for nucleophilic radiofluorination were prepared in three steps from commercially available starting materials (Figure 2). 2-Phenylbenzo[*d*]thiazole **2** was synthesized by reacting 2-aminobenzenethiol with benzoic acid in the presence of polyphosphoric acid.^22^*N*-Alkylation of 2-phenylbenzo[*d*]thiazole **2** took place with 3-iodopropyl trifluoromethanesulfonate or 3-fluoropropyl trifluoromethanesulfonate to yield the triflate salts **3** and **4**, which were subsequently reduced with sodium borohydride to obtain the nonradioactive reference compound **1** and precursor **5** for ^18^F-labeling.

### Radiosynthesis

**Figure 3:**
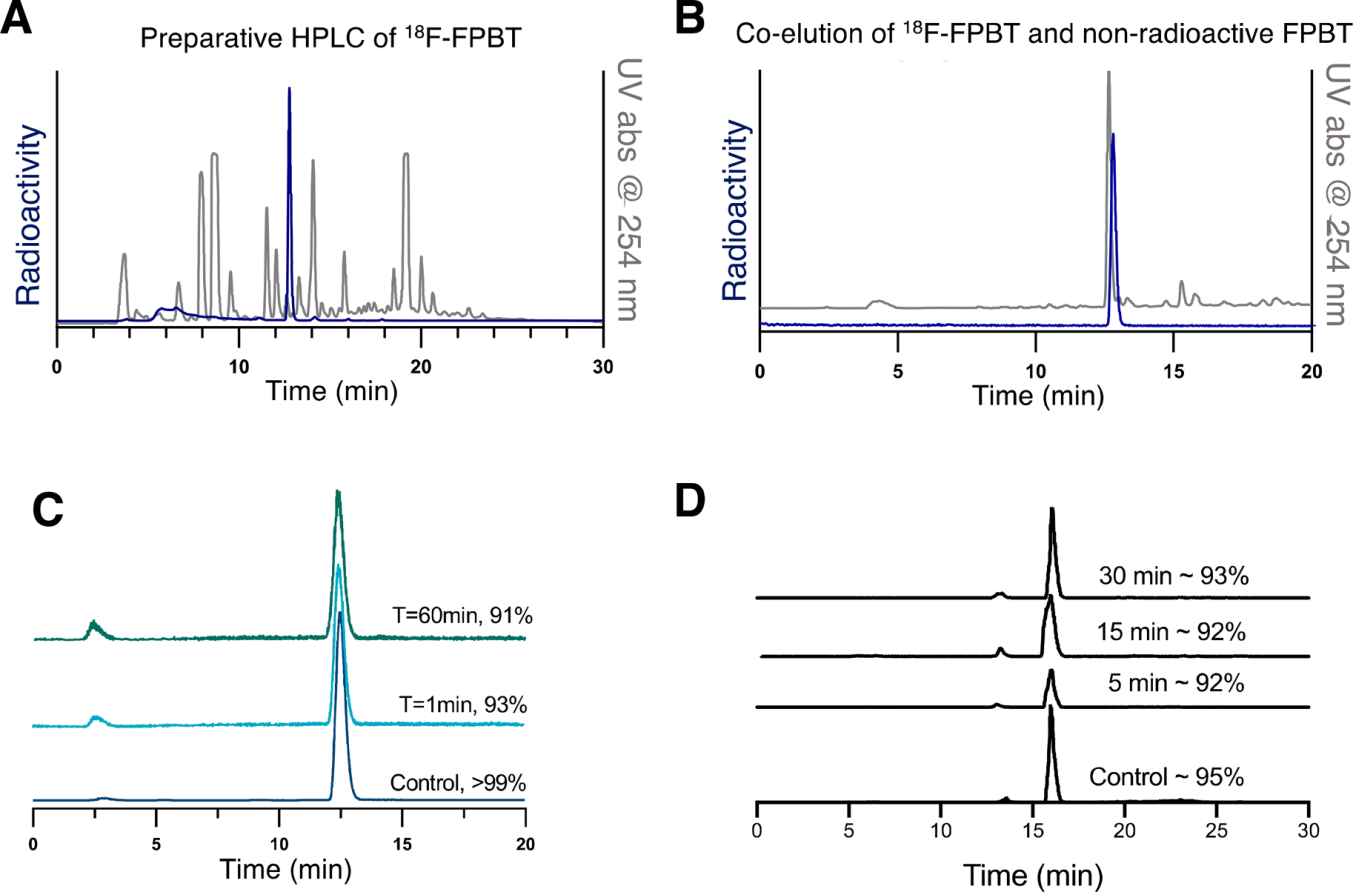
Production and coelution HPLC chromatogram and serum stability. (A) HPLC chromatogram of the crude ^18^F-FPBT radiolabeling reaction mixture. (B) Coelution of purified ^18^F-FPBT with its nonradioactive reference compound **1**. (C, D) Serum stability of ^18^F-FPBT and ^18^F-FPBT-Ox at 37 °C determined by radioHPLC with two different HPLC methods.

^18^F-FPBT was radiosynthesized by a one-step ^18^F-labeling of alkyl iodide precursor **5** with high radiochemical conversion (60 ± 20%, *n* = 10) as indicated by radioHPLC chromatogram of the crude reaction mixture. (Figure 3A) The decay corrected isolated radiochemical yields (RCYs) were consistently around 33 ± 8% (*n* = 10) with >98% radiochemical purity. The manual radiosynthesis, purification, and formulation of ^18^F-FPBT took around 2.5 h from the end of bombardment until formulation. The oxidized analogue, ^18^F-FPBT-Ox, was obtained by reacting ^18^F-FPBT with an excess of potassium superoxide in PBS in 15 min and isolated with >90% radiochemical purity. The identity of both ^18^F-labeled compounds was confirmed by HPLC coelution with the corresponding nonradioactive reference compounds (Figures 3B and Supplementary Figure 1). The molar activity of ^18^F-FPBT was measured as 168 ± 39 GBq/μmol (*n* = 3) when starting with around 1.2 GBq of ^18^F-fluoride.

### Lipophilicity Measurements

^18^F-FPBT has a Log*D* of 1.00 ± 0.08 (*n* = 6), and its oxidized form ^18^F-FPBT-Ox has a significantly lower Log*D* of −1.00 ± 0.04 (*n* = 6) determined using a variation of the conventional shake-flask method.

### *In Vitro* Stability of ^18^F-FPBT and ^18^F-FPBT-Ox

^18^F-FPBT is stable in both pure ethanol and 5% ethanol in PBS in the presence of ascorbic acid (0.01 mg/mL) for 4 h (not tested for longer). When incubated in rat serum at 37 °C, about 90% of ^18^F-FPBT was intact in 1 h (Figure 3C). The ^18^F-FPBT-Ox also exhibits excellent serum stability (Figure 3D).

### *In Vitro* Reactivity of ^18^F-FPBT toward Various ROS

**Figure 4:**
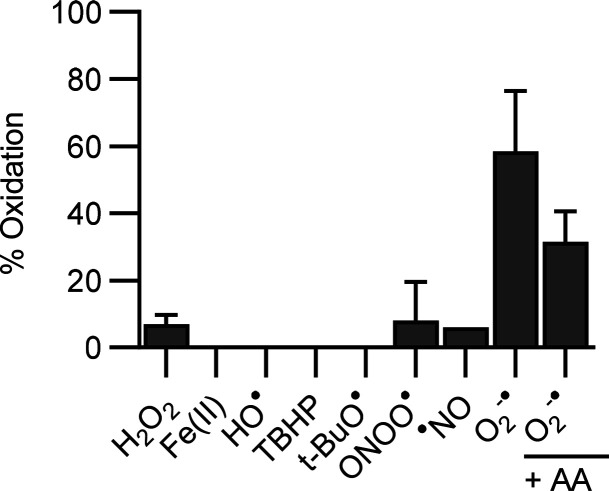
Chemoselectivity of ^18^F-FPBT. Reactivity of ^18^F-FPBT to different oxidants (O_2_^–^•, superoxide; H_2_O_2_, hydrogen peroxide; Fe(II), iron(II); HO•, hydroxyl radical; TBHP, *tert*-butyl hydroperoxide; *t*-BuO•, *tert*-butoxy radical; ONOO•, peroxynitrite; •NO, nitric oxide; AA, ascorbic acid); data represent the mean of 2–4 experiments ± SEM.

^18^F-FPBT showed rapid (in 5 min) and selective oxidation 58 ± 8% (*n* = 4) by superoxide, which can be partially inhibited to 31 ± 5% (*n* = 3) in the presence of ascorbate (1 mg/mL) (Figure 4). In contrast, ^18^F-FPBT had little or no reactivity to other biologically relevant ROS.

### ^18^F-FPBT *In Vivo* PET/CT Imaging of Healthy Mice

^18^F-FPBT had fast blood clearance. It was rapidly taken up and cleared by the heart, lungs, spleen, and brain with low nonspecific background retention in these organs. The radiotracer is excreted by both renal and hepatobiliary routes in less than 1 h (Supplementary Figure 3).

### Post-Mortem Biodistributions in Healthy Rats

**Figure 5:**
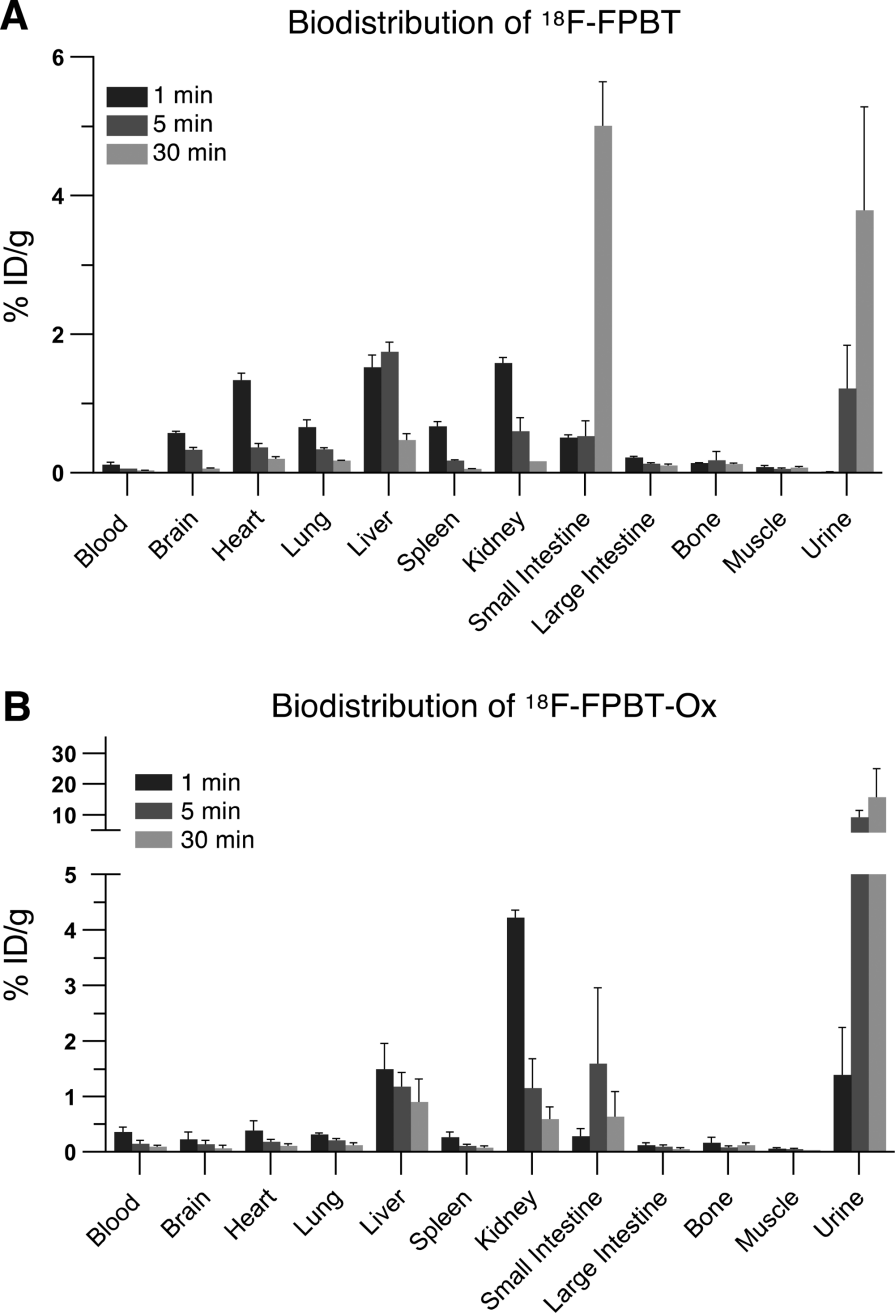
Biodistribution of ^18^F-FPBT and ^18^F-FPBT-Ox in healthy rats. *Ex vivo* tissue biodistribution of ^18^F-FPBT (A) and ^18^F-FPBT-Ox (B) in healthy Wistar rats at 1, 5, and 30 min after intravenous injection. Data represented as mean ± SEM (*n* = 3/group).

*Ex vivo* biodistribution studies in healthy Wistar rats also showed rapid uptake of ^18^F-FPBT by major organs, including the brain, heart, lung, and spleen, and fast clearance from the blood pool at 1 min postinjection (Figure 5A). ^18^F-FPBT was excreted by both renal and hepatobiliary routes. Conversely, ^18^F-FPBT-Ox shows significantly reduced uptake in major organs and was quickly cleared by the kidneys and excreted through the urine (Figure 5B).

### Rat Model of Doxorubicin-Induced Cardiotoxicity

**Figure 6:**
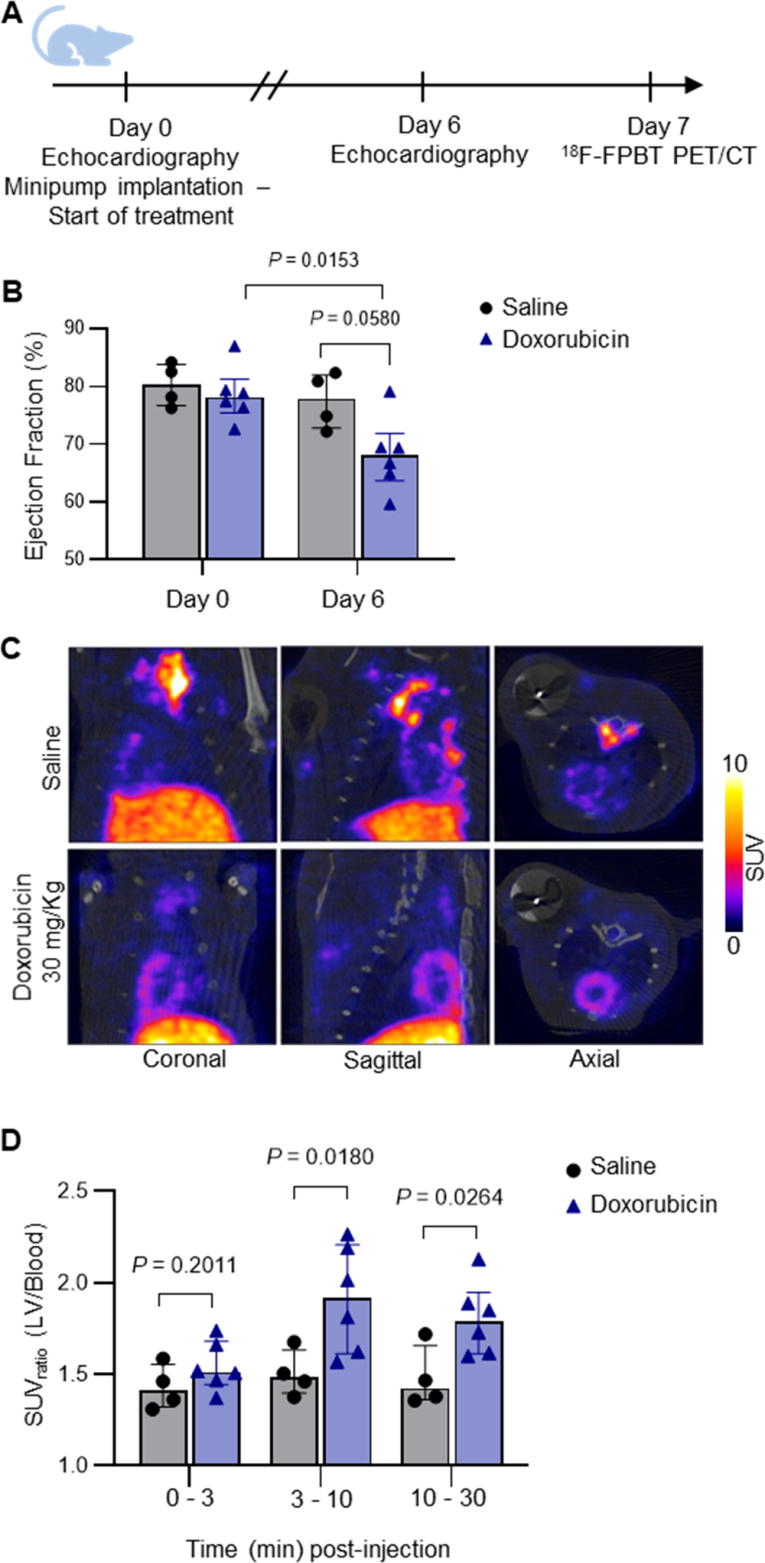
PET/CT imaging of ^18^F-FPBT in a rat model of doxorubicin-induced cardiotoxicity. (A) Experimental design and timeline. (B) Ejection fraction for control (saline, *n* = 4) and treated (doxorubicin, *n* = 6) Wistar rats before and after treatment. (C) Representative sagittal, coronal, and axial images (3–10 min) from coregistered PET/CT imaging of ^18^F-FPBT in Wistar rats, following 7 day exposure to saline (*n* = 4, top) or doxorubicin (*n* = 6, bottom). (D) SUVR _LV/blood_ between 0 and 3, 3–10, and 10–30 min postinjection of ^18^F-FPBT. Data represented at the median ± interquartile range.

Either doxorubicin or saline was delivered to two groups of rats via osmotic pumps to develop the animal model of doxorubicin-induced cardiotoxicity and the control group. From the day of minipump implantation to the day of imaging (7 days), animals in the saline control group maintained their weight with small variations [1.3 ± 2.5% (*n* = 4) increase], while doxorubicin-treated animals consistently lost weight [16.2 ± 8.9% (*n* = 6) decrease]. Rats treated with doxorubicin showed a statistically significant reduced (15%) left ventricular ejection fraction at day 6 compared to their baseline values at day 0. However, echocardiography cannot detect the statistical difference of the left ventricular ejection fraction between doxorubicin-treated animals and their time-matched vehicle controls at day 6 (Figure 6B).

### PET/CT Imaging in Rat Model of Doxorubicin-Induced Cardiotoxicity

A dynamic PET/CT scan (30 min) was performed on the rats that received either doxorubicin or saline. The PET data of each animal was reconstructed into three frames: 0–3, 3–10, and 10–30 min. The standard uptake value ratio (SUVR) between the left ventricle and blood was calculated to determine the radioactivity retention in the heart of each animal. The doxorubicin-treated rats showed higher myocardial radioactivity retention than the vehicle control group in the PET imaging (Figure 6C). The SUVR_LV/blood_ of the doxorubicin-treated rats was statistically significantly (*p* < 0.05) higher than the vehicle control group (Figure 6D). In the time–activity curve extracted from PET data (Supplementary Figure 4a,b), the median myocardial SUV values of the doxorubicin-treated animals are higher than their median blood SUV values in the 30 min PET scan, while the median myocardial and blood SUV values of the saline-treated control group are very similar at all of the time points. In addition, the median SUVR_LV/blood_ of doxorubicin-treated animals is also higher than the saline control group in the 30 min PET scan (Supplementary Figure 4c).

### *In Vivo* Blood Stability of ^18^F-FBPT-Ox

Subsequently, we examined the *in vivo* blood stability of ^18^F-FPBT-Ox at 30 min post IV injection. Plasma proteins were precipitated, and the supernatant was analyzed by radio-HPLC. The HPLC eluent was collected every minute in 30 vials separately and submitted to gamma counting. The counts per minute in each vial were plotted against the corresponding time point. Increased gamma counts (Supplementary Figure 5) were only observed very close to the HPLC retention time of the ^18^F-FPBT-Ox (Figure 3D).

### *Ex Vivo* Heart Biomarkers of Oxidative Stress

Cardiac oxidative stress biomarkers including glutathione, maldonaldehyde, mitochondrial iron, and cytosolic iron were measured following the established literature methods. The levels of these biomarkers were marginally higher in the doxorubicin-treated group comparing to the saline control group (Supplementary Figure 6).

## Discussion

The radiosynthesis of ^18^F-FPBT was achieved in a late-stage one-step radiolabeling with sufficiently high yield to make it suitable for translation into a fully automated radiosynthesizer such as GE FASTlab. We systematically investigated the reactivity and chemoselectivity of ^18^F-FPBT with various biorelevant ROS *in vitro* and observed rapid and selective oxidation by superoxide, which can be significantly inhibited by a large excess of ascorbic acid. We suspect the redox potential, half-life, and the steric hindrance of the ROS played a combination role in the chemoselectivity of ^18^F-FPBT toward various ROS oxidation. The redox potential of hydroxyl radical (2.33 *E*°/V) is significantly higher than superoxide (0.94 *E*°/V).^23^ However, the hydroxyl radical has a much shorter half-life (10^–10^ s) than that of the superoxide (10^–6^ s).^24^ Therefore, ^18^F-FPBT appears to be inert to hydroxyl radical but reactive toward superoxide. Such chemoselectivity of 2-benzothiazolines toward superoxide rather than hydroxyl radical has also been observed by others. For example, Zhang has reported that the 2-(2-pyridil)-benzothiazoline was oxidized by superoxide to form 2-(2-pyridil)-benzothiazole but not by the hydroxyl radical.^20^ This might also be the reason why ^18^F-FPBT has shown some reactivity toward other longer half-life ROS such as nitric oxide (a few seconds), peroxynitrite (10^–3^ s), and hydrogen peroxide (stable) despite the fact that they have a much lower redox potential than the hydroxyl radical.^24^ On the other hand, an alkylperoxyl radical (∼1.0 *E*°/V) such as a *tert*-butoxy radical has a similar redox potential as the superoxide (0.94 *E*°/V) but has a significantly higher steric hindrance.^23^ This could explain that no oxidation was observed when ^18^F-FPBT was treated with a *tert*-butoxy radical. The X-ray structure of 2-phenylbenzothiazoline indicates that the phenyl ring is perpendicular to the benzothiazoline, which increases the steric hindrance of C2 proton from oxidant attack.^19^

Next, the Log*D* values of ^18^F-FPBT and ^18^F-FPBT-Ox were determined to be around 1 and −1, respectively. This raises the possibility that the lipophilic ^18^F-FPBT can penetrate the cell membrane and react with intracellular superoxide. After oxidation, the hydrophilic ^18^F-FPBT-Ox would be trapped inside the cells and generate contrast for PET imaging of oxidative stress. The biodistribution of both compounds in healthy rats demonstrated that ^18^F-FPBT has rapid uptake by all major organs including the brain. Conversely, ^18^F-FPBT-Ox showed significantly reduced uptake in these organs and was quickly cleared by the kidneys. The PET/CT imaging of ^18^F-FPBT in healthy mice provided further evidence that ^18^F-FPBT has fast uptake and clearance by the heart, lung, and brain, which generates low background for imaging oxidative stress in these organs. Additionally, there was little radioactivity accumulation in the bone by both rats and mice indicating that ^18^F-FPBT is inert toward defluorination *in vivo*.

To assess whether ^18^F-FPBT can detect oxidative stress *in vivo*, PET imaging was performed in a rat model of doxorubicin-induced cardiotoxicity, which is characterized by mitochondrial dysfunction leading to excess intracellular superoxide generation.^11−13^ Increased myocardial radioactivity uptake was observed in the PET images of doxorubicin-treated rats comparing to the saline control group. The doxorubicin-treated animals also had a significantly higher LV to blood SUV ratio than the saline control group in the same period. Post-mortem biodistribution studies at 30 min post IV injection of ^18^F-FPBT did not show an increased uptake in the heart of doxorubicin-treated animals. However, this represents a single late time point and does not account for radiotracer bioavailability. PET/CT imaging overcomes these limitations of sampling bias, and these results give us the confidence that ^18^F-FPBT can be used to detect oxidative stress in cardiac pathologies. Moreover, blood metabolite analysis has shown that ^18^F-FPBT-Ox is stable in blood at least 30 min post IV injection, which further supports the trapping mechanism of ^18^F-FPBT post ROS oxidation.

*Ex vivo* measurements of cardiac biomarkers of oxidative stress (glutathione, malondialdehyde, and iron accumulation) were marginally elevated by doxorubicin treatment, but did not reach statistical significance (Supplementary Figure 6), largely due to the interindividual variability in response, which is a well-characterized aspect of doxorubicin cardiotoxicity, both experimentally and in patients; this phenomenon is, in fact, one of the prime motivators for developing imaging agents that would enable personalized medicine approaches for titrating chemotherapeutic regimes to maximize therapeutic effectiveness while minimizing cardiac risk.

^18^F-FPBT is a novel small molecule probe that can be readily prepared by nucleophilic ^18^F-labeling in one step with good radiochemical conversion. ^18^F-FPBT exhibits chemoselectivity toward superoxide oxidation. The radiotracer was taken up by major organs and excreted through both renal and hepatobiliary routes rapidly. These characteristics would, in principle, make ^18^F-FPBT a good candidate for clinical translation. Although further characterization in additional models of oxidative stress is required, we observed an increased uptake in the hearts of doxorubicin-treated rats when compared to controls, highlighting the potential of ^18^F-FPBT as a PET tracer to noninvasively detect oxidative stress *in vivo*.

## Supporting Information Available

Synthesis and characterization of the nonradioactive reference compound **1**, intermediates, **2**, **3**, **4**, and radiolabeling precursor **5**; HPLC chromatograms of ^18^F-labeled compounds coelution with the corresponding nonradioactive reference compounds and quality control; ^18^F-FPBT *in vivo* PET/CT imaging of healthy mice; time–activity curves of ^18^F-FPBT in rats; blood stability of ^18^F-FPBT-Ox 15 min post intravenous injection; *ex vivo* measurements of cardiac biomarkers of oxidative stress; ^1^H, ^13^C NMR, and ^19^F spectra for all compounds prepared (PDF)
