# Supplementary material for: A Reactivity-Based 18F-Labeled Probe for PET Imaging of Oxidative Stress in Chemotherapy-Induced Cardiotoxicity
Source: Mol Pharm. 2021 Nov 30;19(1):18–25. doi: 10.1021/acs.molpharmaceut.1c00496 (PMC8728736; doi:10.1021/acs.molpharmaceut.1c00496)
Supplement: Supplementary file 1 — mp1c00496_si_003.pdf [file mp1c00496_si_003.pdf]

A reactivity-based ^{18}F-labeled probe for PET imaging of oxidative stress in chemotherapy-induced cardiotoxicity

Filipa Mota[§], Victoria R Pell[§], Nisha Singh[§,†], Friedrich Baark[§], Edward Waters[§], Pragalath Sadasivam[§], Richard Southworth[§*], and Ran Yan[§*]

[§] School of Biomedical Engineering & Imaging Sciences, King's College London, King's Health Partners, St Thomas' Hospital, London, SE1 7EH, United Kingdom;

[†] Department of Neuroimaging, Institute of Psychiatry, Psychology, and Neuroscience, King's College London, London, SE5 8AF, United Kingdom

Supplementary Information

Chemical syntheses

Materials and Methods

^{1}H and ^{13}C NMR spectra were recorded at room temperature on a Bruker Avance 400 instrument operating at the frequency of 400 MHz for ^{1}H and 100 MHz for ^{13}C. Chemical shifts are reported in ppm relative to dimethyl sulfoxide (δ 2.48, m) and coupling constants (*J*) are given in Hertz. HPLC analysis was performed with an Agilent 1200 HPLC system equipped with a 1200 series diode array detector. Radio-HPLC analysis was performed with an Agilent 1200 HPLC system equipped with a series diode array detector and Raytest GABI Star radioactivity detector. ^{18}F-Fluoride was purchased from either the PET Center at St. Thomas' Hospital or Alliance Medical UK. All reagents were purchased from Sigma-Aldrich and were used without further purification. Data are represented as mean ± SD. Chromatographs and graphs were plotted on GraphPad Prism 8.

Synthesis of compound 2

2-Phenylbenzo[d]thiazole ***2***[(1)]**:** A mixture of benzoic acid (1.50 g, 12.3 mmol, 1.4 equiv.), 2-aminothiophenol (0.99 mL, 9.5 mmol, 1.0 equiv.) and polyphosphoric acid (4.50 g, 3 x acid weight) was heated with stirring at 150 °C overnight. Upon cooling, the resulting mixture was neutralised with 7 % NH_4OH (25 mL) and stirred for 2 hrs. The green solid was filtered and thoroughly rinsed with 7 % NH_4OH solution. The precipitate was recrystallized from a mixture of diethyl ether and hexane to give the title compound (1.34 g, 67 % yield). ^{1}H NMR (400 MHz, DMSO-d_6) δ: 8.19 – 8.05 (4H, m, Ar-*H*), 7.63 – 7.53 (4H, m, Ar-*H*), 7.48 (1H, ddd, *J* = 8.3, 7.2, 1.2 Hz, Ar-*H*); ^{13}C NMR (100 MHz, DMSO-d_6) δ: 167.25 (*qC*), 153.50

(*qC*), 134.40 (*qC*), 132.79 (*qC*), 131.41 (*C*H), 129.39 (*C*H), 127.16 (*C*H), 126.65 (*C*H), 125.53 (*C*H), 122.86 (*C*H), 122.36 (*C*H); HRMS: calculated for $[M+H]^+$ $C_{13}H_{10}NS$ 212.0534, found m/z 212.0522

Synthesis of compound 3

3-(3-Fluoropropyl)-2-phenylbenzo[d]thiazol-3-ium trifluoromethanesulfonate ***3***: 3-fluoropropyl trifluoromethanesulfonate was prepared *in situ* following a modified published protocol.[(2)] To a solution of trifluoromethanesulfonic anhydride (5.0 mL, 30 mmol) in anhydrous DCM (15 mL) was added a solution of 3-fluoro-1-propanol (2.4 mL, 30 mmol) and anhydrous pyridine (2.4 mL, 30 mmol) in anhydrous DCM (20 mL) dropwise at 0 ^{o}C under nitrogen. A white precipitate of pyridinium salt was formed upon addition. The reaction mixture was stirred at 0 ^{o}C for 30 min before quenching with deionised water (20 mL). The organic layer was separated and washed with deionised water (50 mL x 2) and brine (50 mL), dried over $MgSO_4$, and concentrated in vacuum to give 3-fluoropropyl trifluoromethanesulfonate as a light brown liquid which was used for next step without further purification. 3-Fluoropropyl trifluoromethanesulfonate (525.4 mg, 2.5 mmol, 5.0 equiv.) was added to a suspension of $NaHCO_3$ (210.0 mg, 2.5 mmol, 5.0 equiv.) and **2** (105.6 mg, 0.5 mmol, 1.0 equiv.) in nitrobenzene (10 mL). The reaction mixture was stirred at room temperature for 24 hours after which it was purified by flash column chromatography. The reaction mixture was loaded directly onto the column and eluted with DCM until all nitrobenzene was removed. The crude product on the column was then eluted with DCM/MeOH (9:1). The solvents were removed in vacuum to give the quaternary ammonium trifluoromethanesulfonate salt **3** (42.0 mg, 20 % yield). ^{1}H NMR (400 MHz, DMSO-d_6) δ 8.51 (1H, dd, *J* = 8.2, 1.2 Hz, Ar-*H*), 8.43 (1H, d, *J* = 8.6 Hz, Ar-*H*), 7.95 (1H, ddd, *J* = 8.6, 7.3, 1.3 Hz, Ar-*H*), 7.90 – 7.69 (6H, m, Ar-*H*), 4.78 – 4.70 (2H, m, CH_2), 4.49 – 4.33 (2H, m, CH_2), 2.30 – 2.14 (2H, m, CH_2); ^{13}C NMR (100 MHz, DMSO-d_6) δ: 167.8, 150.1, 133.3, 131.2, 129.7, 129.0, 124.4, 67.7 58.2, 33.1; HRMS calculated for $[M]^+$ $C_{16}H_{15}FNS^+$ 272.0909, found m/z 272.0894.

Synthesis of compound 4

3-(3-Iodopropyl)-2-phenylbenzo[d]thiazol-3-ium trifluoromethanesulfonate ***4***: 3-Iodopropyl trifluoromethanesulfonate was prepared *in situ* following a modified published protocol.[(2)] To a solution of trifluoromethanesulfonic anhydride (5.0 mL, 30 mmol) in anhydrous DCM (15 mL) was added a solution of 3-iodo-1-propanol (2.9 mL, 30 mmol) and anhydrous pyridine (2.4 mL, 30 mmol) in anhydrous DCM (20 mL) dropwise at 0 ^{o}C under nitrogen. A white precipitate of pyridinium salt was formed upon addition. The reaction mixture was stirred at 0 ^{o}C for 30 min before quenching with deionised water (20 mL). The

organic layer was separated and washed with deionised water (50 mL x 2), brine (50 mL), dried over $MgSO_4$, and concentrated in vacuum to give 3-iodopropyl trifluoromethanesulfonate as a light brown liquid which was used for next step without further purification. 3-Iodopropyl trifluoromethanesulfonate (795.1 mg, 2.5 mmol, 5.0 equiv.) was added to a suspension of $NaHCO_3$ (210.0 mg, 2.5 mmol, 5.0 equiv.) and **2** (105.6 mg, 0.5 mmol, 1.0 equiv.) in nitrobenzene (10 mL). The reaction mixture was stirred at room temperature for 24 hours after which it was purified by flash column chromatography. The reaction mixture was loaded directly onto the column and eluted with DCM until all nitrobenzene was removed. The crude product on the column was then eluted with DCM/MeOH (9:1). The solvents were removed in vacuum to give the quaternary ammonium trifluoromethanesulfonate salt **4** (84.7 mg, 32 % yield). ^{1}H NMR (400 MHz, DMSO-d_6) δ: 8.59 (1H, dd, *J* = 8.2, 1.2 Hz, Ar-*H*), 8.51 (1H, d, *J* = 8.5 Hz, Ar-*H*), 8.03 (1H, ddd, *J* = 8.5, 7.2, 1.2 Hz, Ar-*H*), 7.97 – 7.76 (6H, m, Ar-*H*), 4.81 – 4.70 (2H, m, CH_2), 3.28 (2H, t, J = 6.7 Hz, CH_2), 2.43 – 2.31 (2H, m, CH_2); ^{13}C NMR (100 MHz, DMSO-d_6) δ: 175.5 (*qC*), 141.8 (*qC*), 134.0 (*C*H), 130.9 (*qC*), 130.5 (*C*H), 130.3 (*C*H), 129.1 (*C*H), 125.7 (q*C*), 125.3 (*C*H), 118.1 (*C*H), 52.2 (*C*H_2), 32.2 (CH_2), 3.06 (CH_2); HRMS: calculated for $[M]^+$ $C_{16}H_{15}INS^+$ 379.9970, found m/z 379.9961.

Synthesis of compound 1

3-(3-Fluoropropyl)-2-phenyl-2,3-dihydrobenzo[d]thiazole ***1*****:** 3-(3-Fluoropropyl)-2-phenylbenzo[d]thiazol-3-ium trifluoromethanesulfonate **3** (92.6 mg, 0.22 mmol) was dissolved in a solution of THF (5 mL) and MeOH (10 mL) in a round-bottom flask covered in aluminium foil. $NaBH_4$ (8.0 mg, 0.22 mmol) in MeOH (1 mL) was added dropwise to the brown solution of **3** and the resulting mixture was stirred for 20 minutes during which time the solution turned colorless. The solvents were removed in vacuum. The resulting solid was re-dissolved in DCM (20 mL) and washed with water (10 mL). The water layer was extracted with additional DCM (10 mL x 2). The combined organic phase was washed with brine and dried over anhydrous $MgSO_4$. The solvent was removed under reduced pressure and the crude material was purified by flash column chromatography using a gradient of 0 – 30 % ethyl acetate in hexane to give the title compound (31.2 mg, 52 % yield). ^{1}H NMR (400 MHz, DMSO-d_6) δ 7.58 – 7.52 (2H, m, Ar-C*H*), 7.48 – 7.40 (3H, m, Ar-C*H*), 7.11 – 7.01 (2H, m, Ar-C*H*), 6.70 (1H, td, *J* = 7.5, 1.1 Hz, Ar-C*H*), 6.58 (1H, dd, *J* = 8.0, 1.1 Hz, Ar-C*H*), 6.41 (1H, s, C*H*), 4.59 – 4.38 (2H, m, CH_2), 3.18 – 2.91 (2H, m, CH_2), 1.97 – 1.71 (2H, m, CH_2); ^{13}C NMR (100 MHz, DMSO-d_6) δ 146.6 (q*C*), 140.6 (qC), 128.8 (*C*H), 128.7 (*C*H), 126.9 (*C*H), 125.8 (*C*H), 124.6 (q*C*), 121.2 (*C*H), 118.6 (*C*H), 107.1 (*C*H), 82.55 and 80.94 (CH_2F), 72.2 (*C*H), 42.2 (CH_2), 26.5 (CH_2); ^{19}F NMR (376 MHz, DMSO-d_6) δ -106.93; HRMS: calculated for $[M\text{-}H]^+$ $C_{16}H_{15}FNS$+ 272.0909, found m/z 272.0895 (oxidised compound).

Synthesis of compound 5

3-(3-Iodopropyl)-2-phenyl-2,3-dihydrobenzo[d]thiazole ***5*****:** 3-(3-Iodopropyl)-2-phenylbenzo[d]thiazol-3-ium trifluoromethanesulfonate **4** (116.4 mg, 0.22 mmol) was dissolved in a solution of THF (5 mL) and MeOH (10 mL) in a round-bottom flask covered in aluminium foil. $NaBH_4$ (8.0 mg, 0.22 mmol) in MeOH (1 mL) was added dropwise to the brown solution of **5** and the resulting mixture was stirred for 20 minutes during which time the solution turned colorless. The solvents were removed in vacuum. The resulting solid was re-dissolved in DCM (20 mL) and washed with water (10 mL). The water layer was extracted with additional DCM (2 x 10 mL). The combined organic phase was washed with brine and dried over anhydrous $MgSO_4$. The solvent was removed under reduced pressure and the crude material was purified by flash column chromatography using a gradient of 0 – 30 % ethyl acetate in hexane to give the title compound (47 mg, 56 % yield). ^{1}H NMR (400 MHz, DMSO-d_6) δ 7.60 – 7.52 (2H, m, Ar-C*H*), 7.51 – 7.40 (3H, m, Ar-C*H*), 7.14 – 7.01 (2H, m, Ar-C*H*), 6.76 – 6.68 (1H, m, Ar-C*H*), 6.64 (1H, d, *J* = 7.9 Hz, Ar-C*H*), 6.41 (1H, s, C*H*), 3.27 (2H, q, *J* = 7.0 Hz, Ar-CH_2), 3.02 (2H, ddd, *J* = 14.6, 8.8, 5.8 Hz, CH_2), 2.10 – 1.90 (2H, m, CH_2); ^{13}C NMR (100 MHz, DMSO-d_6) δ 146.6 (q*C*), 140.6 (q*C*), 128.8 (*C*H), 128.7 (*C*H), 126.9 (*C*H), 125.8 (*C*H), 124.6 (q*C*), 121.2 (*C*H), 118.6 (*C*H), 107.2 (*C*H), 72.30 (*C*H), 46.60 (*C*H_2), 29.38 (*C*H_2), 4.97 (*C*H_2); HRMS: calculated for $[M+H]^+$ $C_{16}H_{17}INS^+$ 382.0126, found m/z 382.0117.

Supplementary Figures

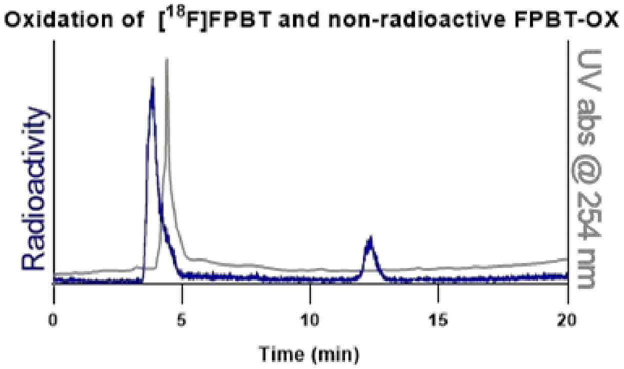

Supplementary Figure 1. HPLC ^{18}F-FPBT-Ox. Oxidation of ^{18}F-FPBT with superoxide observed by radio-HPLC and confirmed with coelution of non-radioactive FPBT-OX. HPLC method: ZORBAX column (300SB-C18, semi-preparative 9.4 X 250 mm, 5 µm) using acetonitrile and water as the mobile phase, at a flow rate of 3.0 mL/min. The following gradient was used: from 50 % to 90 % acetonitrile in 15 min; kept at 90 % acetonitrile for 10 min; from 90 to 50 % acetonitrile in 5 min.

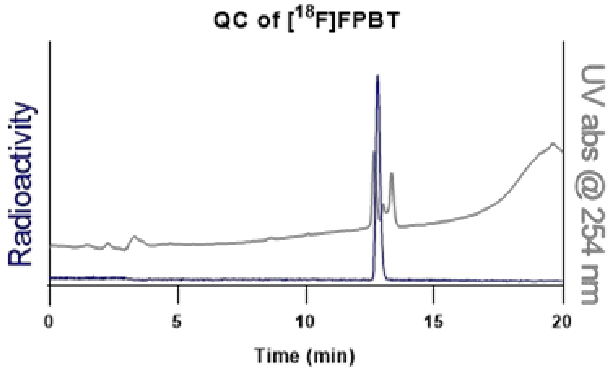

Supplementary Figure 2. HPLC QC analysis of ^{18}F-FPBT. HPLC method: ZORBAX column (300SB-C18, semi-preparative 9.4 X 250 mm, 5 µm) using acetonitrile and water as the mobile phase, at a flow rate of 3.0 mL/min. The following gradient was used: from 50 % to 90 % acetonitrile in 15 min; kept at 90 % acetonitrile for 10 min; from 90 to 50 % acetonitrile in 5 min.

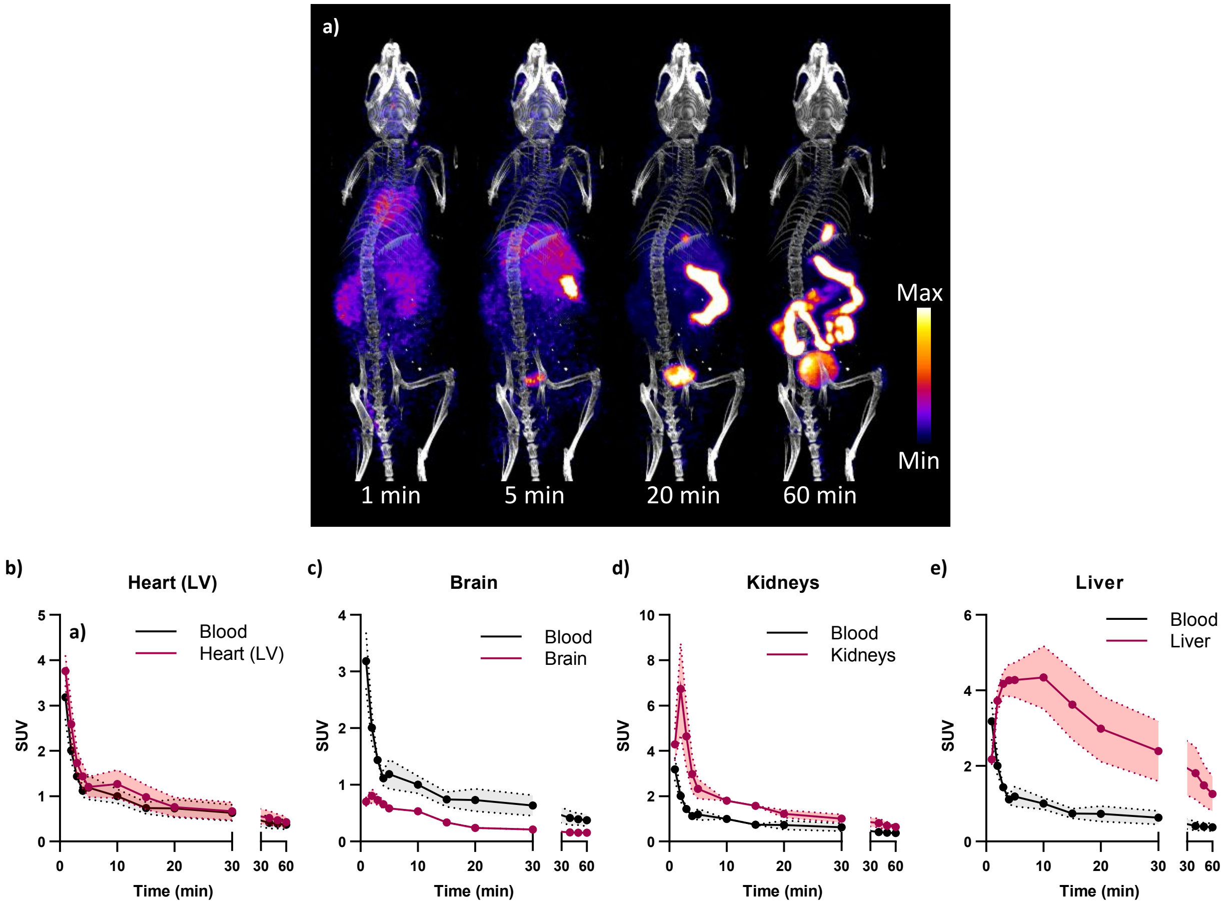

Supplementary Figure 3. PET/CT imaging of ^{18}F-FPBT in C57BL/6 mice. a) Maximum intensity projection PET/CT images of a representative mouse at 1, 5, 20, and 60 min post-injection. ^{18}F-FPBT showed rapid penetration into heart wall (**b**) and brain (**c**), and was excreted by both renal (**d**) and hepatobiliary (**e**) routes. Data represented at SUV mean ± SD, n = 3.

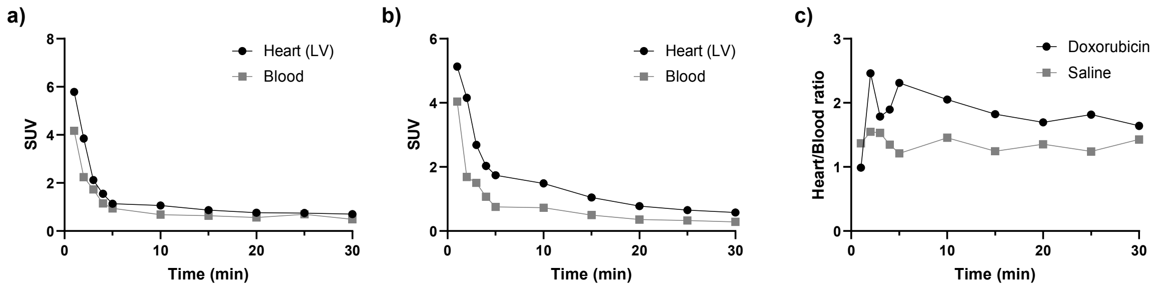

Supplementary Figure 4. Time-activity curves of ^{18}F-FPBT in rats. Time-activity curves in saline control (**a**) and doxorubicin-treated (**b**) rats in the heart and blood pool. (**c**) Heart-to-blood ratios of ^{18}F-FPBT in saline- and doxorubicin-treated rats over time. Data are represented as median. For the time activity curve, the PET data was reconstructed into 1 min per frame in the first 5 min and then 5 minutes per frame from 5 to 30 min.

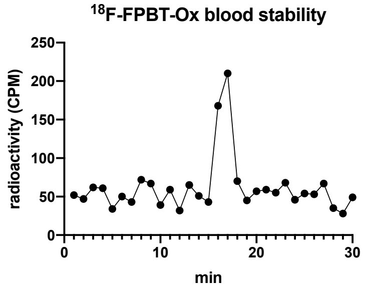

Supplementary Figure 5. Blood stability of ^{18}F-FPBT-Ox 30 min post intravenous injection. HPLC method: ZORBAX column (300SB-C18, semi-preparative 9.4 X 250 mm, 5 µm) Solvent A: H_2O, Solvent B: MeCN; Flow rate: 3.0 mL/min; 0 – 5 min, 0% of B; 5 – 20 min, 0 – 50% of B; 20 – 30 min, 50 – 0% of B. The HPLC eluent was collected every minute in 30 vials separately and submitted to gamma counting. The counts per minute in each vial was plotted against the corresponding time point. Data are represented of two independent experiments.

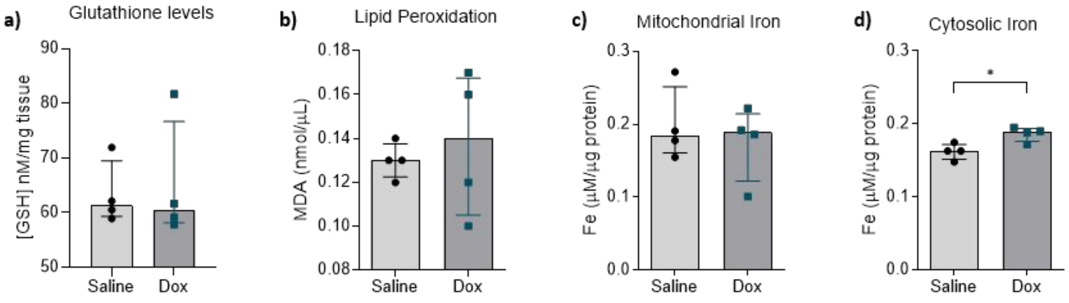

Supplementary Figure 6. *Ex vivo* heart biomarkers of oxidative stress. a) Glutathione, **b)** maldonaldehyde, **c)** mitochondrial iron, and **d)** cytosolic iron levels in the hearts of Doxorubicin-treated (Dox) and saline control rats harvested after the PET imaging. Data are represented as median ± interquartile range, n = 4. * is used to represent p>0.05.

Supplementary Tables

Supplementary table 1. ***Ex vivo*** **biodistribution of ^{18}F-FPBT in healthy rats.** Data are represented as mean % ID/g of tissue ± SD, n = 3.

Organ or tissue	1 min	5 min	30 min
Blood	0.12 ± 0.05	0.06 ± 0.00	0.03 ± 0.00
Brain	0.57 ± 0.04	0.33 ± 0.04	0.06 ± 0.01
Heart	1.33 ± 0.15	0.37 ± 0.08	0.20 ± 0.04
Lung	0.66 ± 0.15	0.34 ± 0.03	0.18 ± 0.01
Liver	1.52 ± 0.25	1.75 ± 0.19	0.47 ± 0.13
Spleen	0.67 ± 0.09	0.18 ± 0.01	0.05 ± 0.01
Kidney	1.58 ± 0.11	0.60 ± 0.27	0.17 ± 0.00
Small Intestine	0.51 ± 0.0.5	0.53 ± 0.31	5.01 ± 0.90
Large Intestine	0.21 ± 0.03	0.13 ± 0.02	0.10 ± 0.03
Bone	0.14 ± 0.01	0.18 ± 0.18	0.13 ± 0.02
Muscle	0.08 ± 0.03	0.05 ± 0.03	0.08 ± 0.02
Urine	0.02 ± 0.01	1.22 ± 0.89	3.79 ± 2.12

Supplementary table 2. ***Ex vivo*** **biodistribution of ^{18}F-FPBT-Ox in healthy rats.** Data are represented as mean % ID/g of tissue ± SD, n = 3.

Organ or tissue	1 min	5 min	30 min
Blood	0.36 ± 0.12	0.15 ± 0.08	0.09 ± 0.04
Brain	0.23 ±0.19	0.14 ± 0.11	0.07 ± 0.07
Heart	0.39 ± 0.25	0.18 ± 0.06	0.11 ± 0.06
Lung	0.31 ±0.05	0.21 ± 0.05	0.12 ± 0.07
Liver	1.49 ± 0.65	1.17 ± 0.37	0.90 ± 0.59
Spleen	0.26 ± 0.13	0.11 ± 0.04	0.07 ± 0.05
Kidney	4.22 ± 0.20	1.15 ± 0.75	0.59 ± 0.31
Small Intestine	0.28 ± 0.20	1.59 ± 1.93	0.63 ± 0.64
Large Intestine	0.12 ± 0.07	0.10 ± 0.04	0.05 ± 0.04
Bone	0.16 ± 0.14	0.09 ± 0.03	0.12 ± 0.06
Muscle	0.06 ± 0.02	0.05 ± 0.02	0.03 ± 0.00
Urine	1.39 ± 1.21	9.29 ± 3.02	15.80 ± 12.98

Supplementary table 3. ***Ex vivo*** **biodistribution of ^{18}F-FPBT in Doxorubicin-treated and control rats 40 min post-injection.** Data are represented as mean % ID/g of tissue ± SD, n = 4 (Saline) and 6 (Doxorubicin).

**Organ or tissue**	**Saline group**	**Doxorubicin group**
Blood	0.07 ± 0.02	0.08 ± 0.06
Heart	0.42 ± 0.17	0.43 ± 0.08
Lung	0.29 ± 0.05	0.31 ± 0.12
Liver	0.65 ± 0.13	0.73 ± 0.24
Spleen	0.11 ± 0.02	0.15 ± 0.07
Kidney	0.42 ± 0.19	0.41 ± 0.14
Small Intestine	3.33 ± 3.38	2.23 ± 1.43
Large Intestine	0.38 ± 0.20	1.42 ±2.07
Bone	0.25 ± 0.22	0.30 ±0.13
Muscle	0.08 ± 0.02	0.10 ± 0.04
Urine	5.25 ± 3.05	12.5 ± 5.33

References:

1. Kil HJ, Lee I-SH. Primary Kinetic Isotope Effects on Hydride Transfer from Heterocyclic Compounds to NAD+ Analogues. *The Journal of Physical Chemistry A.* **2009**;113:10704-10709.

2. Wang J-Q, Kreklau EL, Bailey BJ, Erickson LC, Zheng Q-H. Synthesis and preliminary biological evaluation of O6-[4-(2-[^{18}F] fluoroethoxymethyl) benzyl] guanine as a novel potential PET probe for the DNA repair protein O6-alkylguanine-DNA alkyltransferase in cancer chemotherapy. *Bioorganic & medicinal chemistry.* **2005**;13:5779-5786.

3. Rahman, I.; Kode, A.; Biswas, S. K., Assay for quantitative determination of glutathione and glutathione disulfide levels using enzymatic recycling method. *Nature protocols* **2006,** *1* (6), 3159.

NMR Spectra

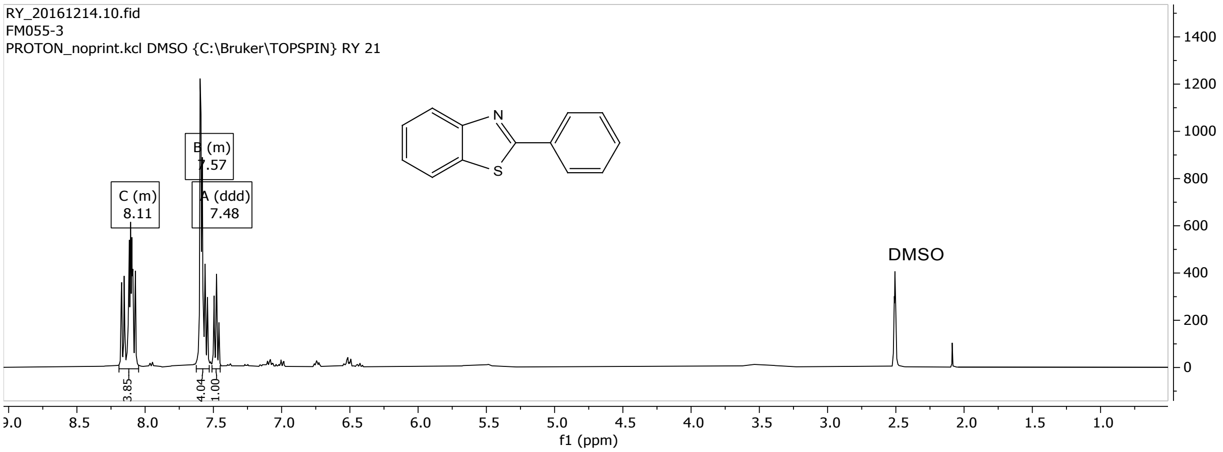

RY_20161214.10.fid
FM055-3
PROTON_noprint.kcl DMSO {C:\Bruker\TOPSPIN} RY 21
B (m) 7.57
C (m) 8.11
A (ddd) 7.48
DMSO
f1 (ppm)

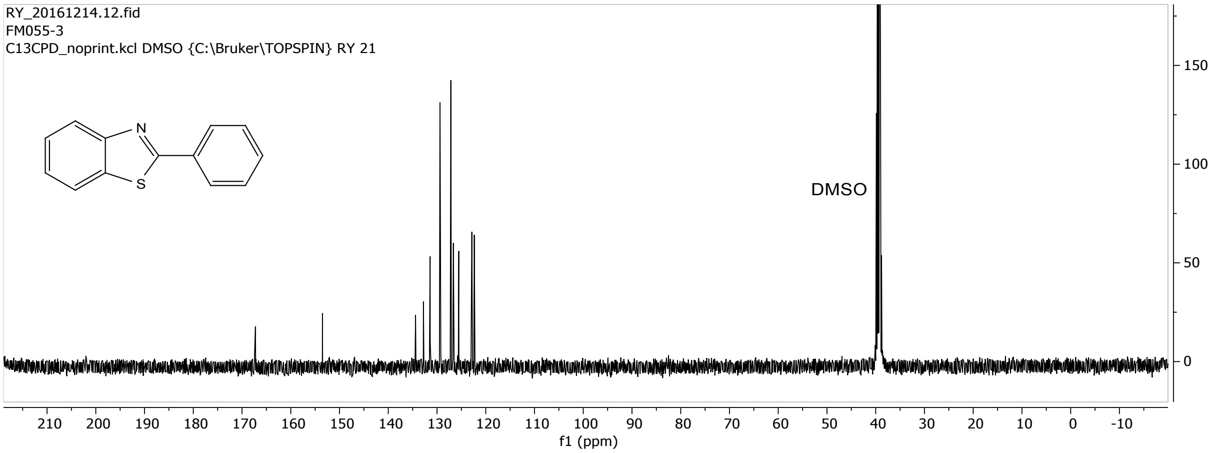

RY_20161214.12.fid
FM055-3
C13CPD_noprint.kcl DMSO {C:\Bruker\TOPSPIN} RY 21
DMSO
f1 (ppm)

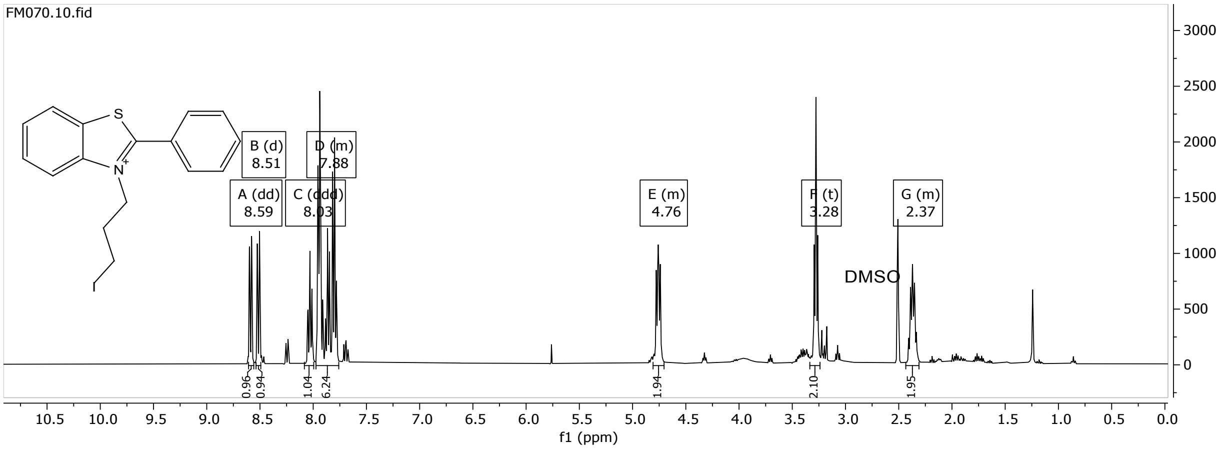

FM070.10.fid
B (d) 8.51
D (m) 7.88
A (dd) 8.59
C (ddd) 8.03
E (m) 4.76
F (t) 3.28
G (m) 2.37
DMSO
f1 (ppm)

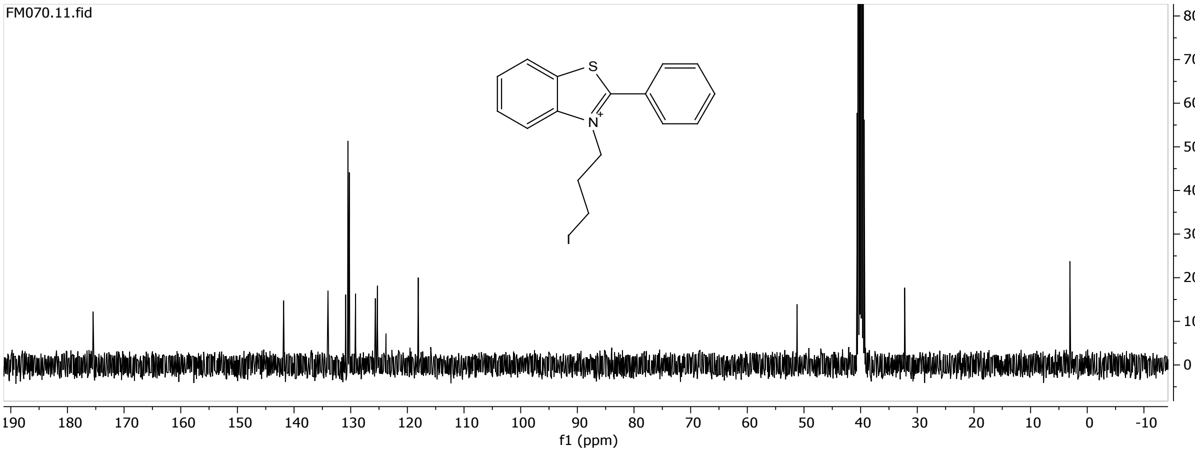
FM070.11.fid
S
N+
I
f1 (ppm)

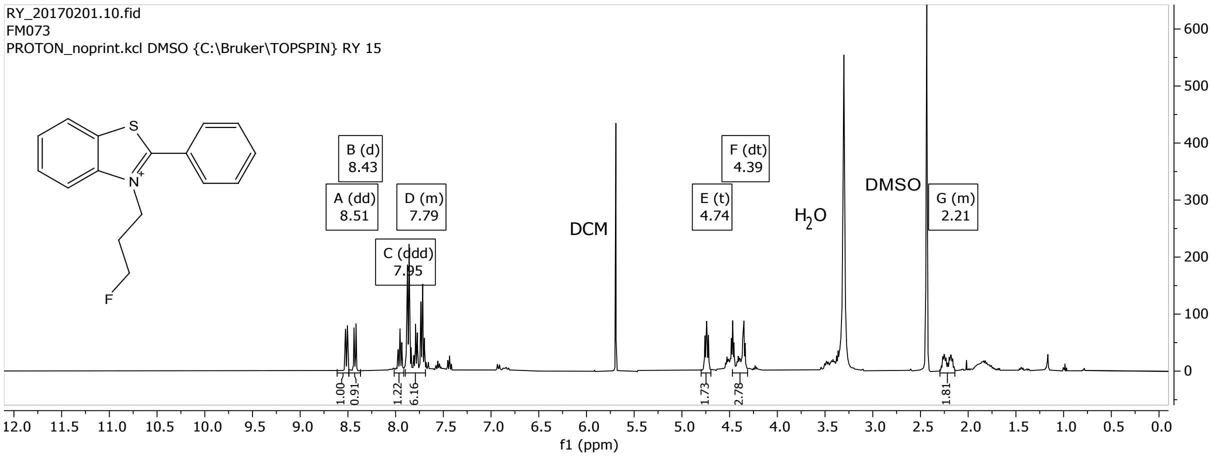
RY_20170201.10.fid
FM073
PROTON_noprint.kcl DMSO {C:\Bruker\TOPSPIN} RY 15
S
N+
F
B (d)
8.43
A (dd)
8.51
D (m)
7.79
C (ddd)
7.95
DCM
F (dt)
4.39
E (t)
4.74
H2O
DMSO
G (m)
2.21
1.00
0.91
1.22
6.16
1.73
2.78
1.81
f1 (ppm)

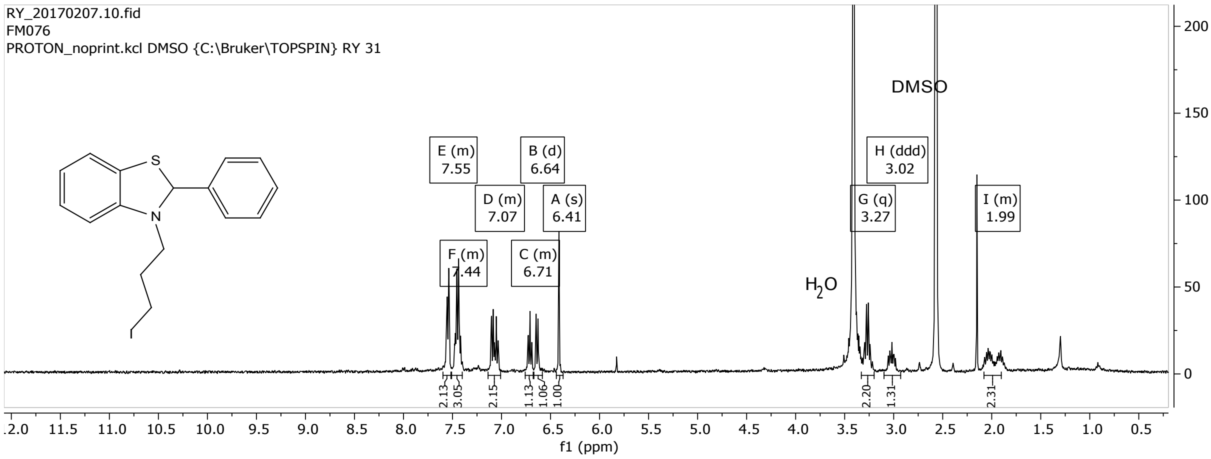
RY_20170207.10.fid
FM076
PROTON_noprint.kcl DMSO {C:\Bruker\TOPSPIN} RY 31
S
N
I
DMSO
E (m)
7.55
B (d)
6.64
H (ddd)
3.02
D (m)
7.07
A (s)
6.41
G (q)
3.27
I (m)
1.99
F (m)
7.44
C (m)
6.71
H2O
2.13
3.05
2.15
1.13
1.06
1.00
2.20
1.31
2.31
f1 (ppm)

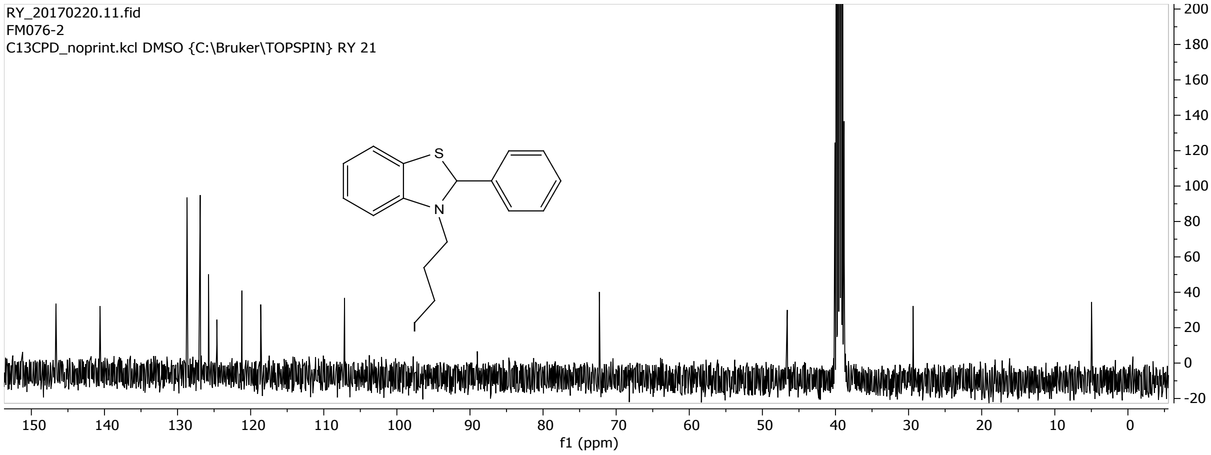
RY_20170220.11.fid
FM076-2
C13CPD_noprint.kcl DMSO {C:\Bruker\TOPSPIN} RY 21
S
N
f1 (ppm)

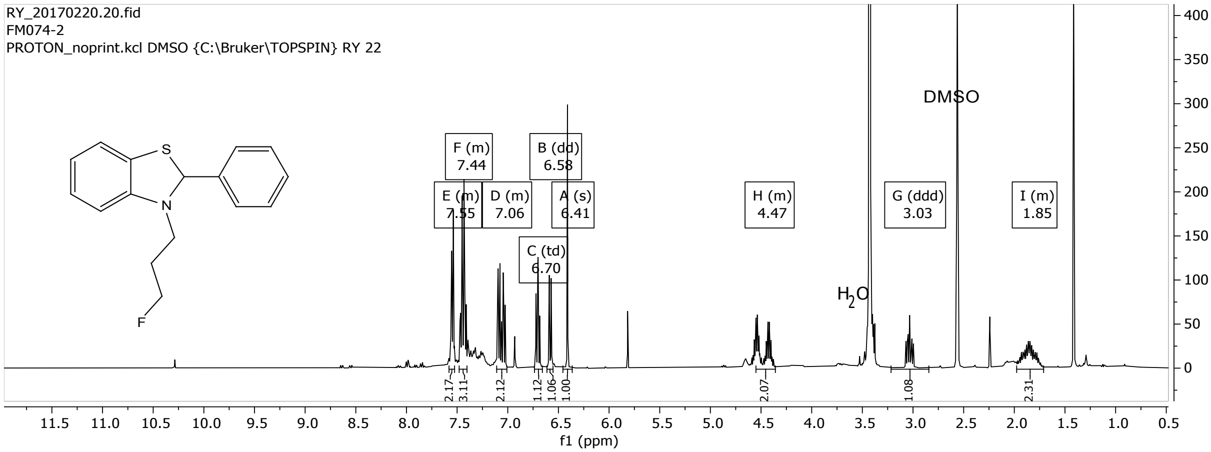
RY_20170220.20.fid
FM074-2
PROTON_noprint.kcl DMSO {C:\Bruker\TOPSPIN} RY 22
S
N
F
DMSO
H2O
F (m) 7.44
B (dd) 6.58
E (m) 7.55
D (m) 7.06
A (s) 6.41
C (td) 6.70
H (m) 4.47
G (ddd) 3.03
I (m) 1.85
2.17
3.11
2.12
1.12
1.06
1.00
2.07
1.08
2.31
f1 (ppm)

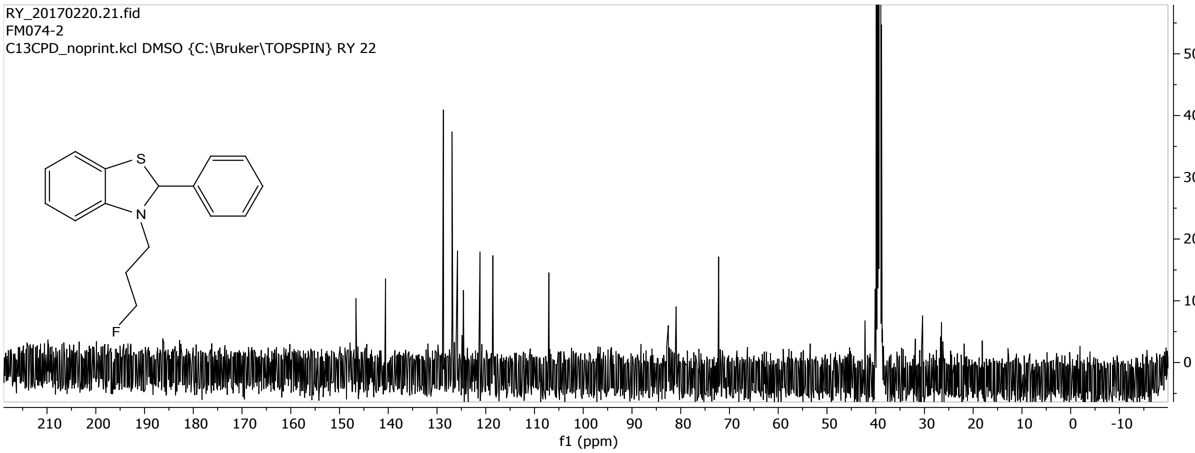
RY_20170220.21.fid
FM074-2
C13CPD_noprint.kcl DMSO {C:\Bruker\TOPSPIN} RY 22
S
N
F
f1 (ppm)

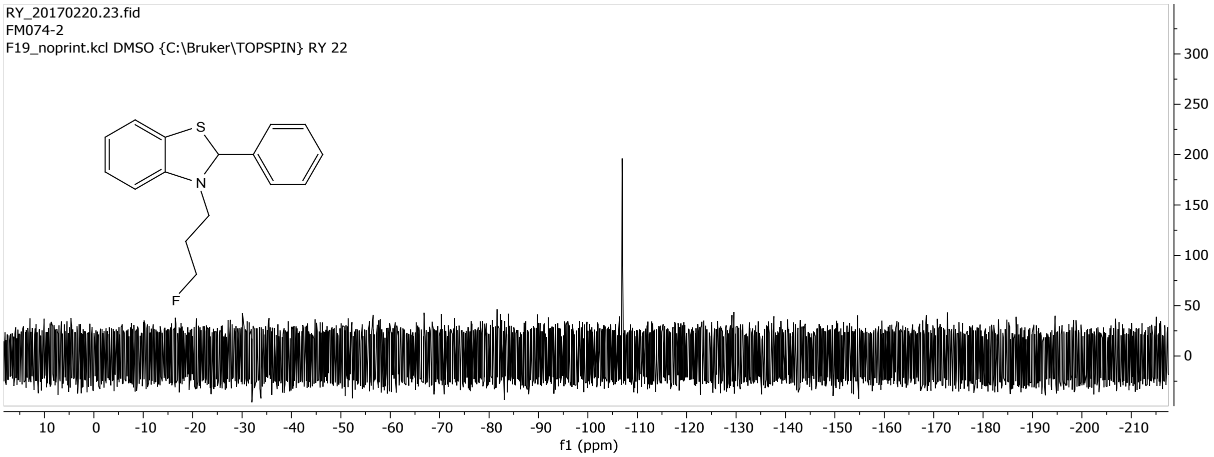
RY_20170220.23.fid
FM074-2
F19_noprint.kcl DMSO {C:\Bruker\TOPSPIN} RY 22
S
N
F
300
250
200
150
100
50
0
10
0
-10
-20
-30
-40
-50
-60
-70
-80
-90
-100
-110
-120
-130
-140
-150
-160
-170
-180
-190
-200
-210
f1 (ppm)
